# Intensity-Based Estimation of Monomeric Brightness for Fluorescent Proteins

**DOI:** 10.3390/ijms262311678

**Published:** 2025-12-02

**Authors:** Michael R. Stoneman, Sanam Bista, Thomas D. Killeen, Ionel Popa, Valerică Raicu

**Affiliations:** Department of Physics and Astronomy, University of Wisconsin-Milwaukee, Milwaukee, WI 53211, USA

**Keywords:** fluorescence microscopy, protein-protein interactions, fluorescence fluctuation spectroscopy, molecular brightness, fluorescent proteins, quantitative fluorescence imaging, protein oligomerization

## Abstract

Fluorescence fluctuation spectroscopy (FFS) techniques rely on determination of monomeric molecular brightness, i.e., the fluorescence intensity of a single, non-aggregated fluorophore, as a critical reference for estimating protein oligomer size. By comparing measured molecular brightness of fluorescently labeled proteins of interest to this monomeric brightness benchmark, FFS enables inference of oligomerization states. However, widely used fluorescent proteins often exhibit self-association, compromising monomeric brightness calibration and introducing errors in brightness-derived oligomer-size estimates. This study compares two strategies for determining monomeric brightness: the conventional fluctuation-based method and a more recently proposed average-intensity-based alternative. The comparison uses two model fluorophores, a fluorescent protein (mCitrine) and the small-molecule dye Janelia Fluor 525 (JF_525_) conjugated to HaloTag. Our results show strong agreement between intensity- and fluctuation-derived brightness values only in the minimally aggregating JF_525_–HaloTag benchmark. In contrast, in the mCitrine samples, where aggregation is more prevalent, only the intensity-based method maintains a consistent estimate across sample preparation, while the fluctuation-based method overestimates brightness when aggregation effects become pronounced. This robustness makes the intensity-based approach a valuable cross-check for monomeric brightness calibration. Our results support a combined strategy, using both methods to improve the reliability of monomeric brightness calibration and protein oligomerization analysis in FFS.

## 1. Introduction

Fluorescence fluctuation spectroscopy (FFS) [1,2,3,4] offers a powerful suite of tools, such as Number and Brightness (N&B) [5,6,7,8], Spatial Intensity Distribution Analysis (SpIDA) [9,10], and Fluorescence Intensity Fluctuation (FIF) [11,12,13], to probe molecular interactions and protein oligomerization in living systems. These techniques rely on measurements of molecular brightness, defined as the average fluorescence intensity per diffusing fluorescent particle within a given excitation volume [14,15,16,17]. Molecular brightness scales linearly with the number of fluorophores in an oligomer, making it an attractive metric for quantifying protein oligomer size. For example, a homodimer tagged with one fluorophore per monomer is expected to produce a molecular brightness approximately twice that of a monomer under otherwise identical conditions.

While FFS-based methods offer several advantages, particularly for live-cell and dilute-solution measurements, they are part of a broader landscape of techniques used to study protein oligomerization. Solution-based methods such as size exclusion chromatography (SEC) [18], analytical ultracentrifugation (AUC) [19], and dynamic light scattering (DLS) [20] provide estimates of hydrodynamic size and aggregation states but generally require high-concentration, purified samples and may lack the resolution to resolve closely spaced oligomeric states. Electrophoretic techniques like native polyacrylamide gel electrophoresis (PAGE) [21] offer qualitative insights into molecular weight but can introduce artifacts related to buffer or detergent conditions. In cellular environments, the organized smooth endoplasmic reticulum (OSER) assay [22] infers self-association through morphological alterations in the endoplasmic reticulum but lacks stoichiometric precision. Methods such as SEC, AUC, DLS, PAGE, or OSER may be more practical for high-concentration purified samples or qualitative screening of self-association, whereas the intensity-based FFS approach is most advantageous when quantifying oligomeric state in live cells or low-concentration environments where molecular brightness readouts are already central to the experimental design. In this context, FFS techniques provide a complementary approach, offering high spatial and temporal resolution with quantitative insight into oligomerization under near-physiological conditions. When calibrated using monomeric controls, molecular brightness becomes a direct proxy for oligomer size: the ratio of observed brightness to a calibrated value of the monomeric molecular brightness, that is, the fluorescence output of a single, non-aggregated fluorophore under the same conditions, reveals the number of proteins comprising the complex, assuming one fluorophore per protein subunit [11,12,23,24,25,26].

The standard route to estimating monomeric brightness is via fluctuation analysis, where fluorescence intensity fluctuations, recorded from a dilute population of monomeric forms of the fluorescent label in solution or embedded within membranes, are used to compute molecular brightness per particle [11,27]. This method has the obvious advantage of being the same analytical framework applied to probe the oligomerization in biological samples of interest. However, this method is not immune to artifacts: even trace amounts of dimers or higher-order aggregates in a nominally monomeric preparation can elevate the apparent molecular brightness. Fluorescent proteins such as EGFP and Citrine are prone to self-association, even in their mostly “monomeric” forms [28,29] (e.g., mCitrine, the A206K mutant form of Citrine). This propensity for dimerization or higher-order oligomerization can result in an overestimated monomeric brightness value, leading to underestimation of oligomeric size in samples with unknown properties.

To address the limitations of fluctuation-based calibration of monomeric brightness, we explore an alternative approach: estimating monomeric molecular brightness, ε, from solution-based measurements using the rather straightforward relation I=CεV, where *I* represents the average fluorescence intensity, *C* the fluorophore concentration (in molecules per volume), and V the effective observation volume from which the signal arises [30], such that ε has units of fluorescence intensity per molecule. In practice, this involves plotting fluorescence intensity as a function of fluorophore concentration and extracting the slope of the resulting line [11,31,32]. If a value for V can be determined, molecular brightness ε can be extracted directly by dividing the slope of the linear fit by V. However, under focused excitation, common to laser-scanning microscopes, this calculation is complicated by the Gaussian nature of the beam. Molecules experience varying excitation intensities depending on their position in the beam, and the boundaries of the excitation field are ill-defined [14,15]. To overcome these challenges, we adopt the concept of a virtual observation volume: a hypothetical region that, if uniformly illuminated at the beam’s peak intensity, would produce the same total fluorescence signal as the real, spatially varying excitation field [14,15,30]. This construct allows us to apply the intensity–concentration–volume framework using a physically meaningful estimate of V. The underlying mathematical model is described in Section 4.3.

In this study, we comparatively evaluate the average-intensity-based approach to estimating monomeric molecular brightness and the traditional intensity fluctuation-based method. We begin by empirically calibrating the effective excitation volume using sub-diffraction size fluorescent nanobeads. The observation volume, denoted $V_{PSF}$, provides a physical basis for connecting average intensity measurements to absolute molecular brightness, and serves as a conceptual bridge between fluctuation- and intensity-derived analyses. We then apply both methods to mCitrine under uninhibited aggregation conditions and show that the two approaches diverge significantly in their monomeric brightness estimates. Under highly controlled preparation conditions, which are labor-intensive and often better reserved for samples containing the biological system of interest, this discrepancy is significantly reduced but not eliminated, highlighting the underlying sensitivity of fluctuation-derived brightness to sample handling. Finally, we evaluate both methods using Janelia Fluor 525 (JF_525_) conjugated to HaloTag [33], a small-molecule labeling system expected to exhibit reduced aggregation compared with fluorescent proteins [34,35]. In this benchmark system, the two approaches yield closely matching brightness estimates, consistent with a truly monomeric population.

The results presented in this manuscript establish that the intensity-based determination of monomeric brightness, when paired with careful volume and concentration measurement, offers a robust and interpretable path to monomeric brightness estimation based on separate knowledge of the fluorophore concentration and the effective observation volume. Accurate measurement of both these quantities is straightforward in solution-phase experiments but may be difficult or impossible in spatially heterogeneous environments such as membranes or organelles. However, as we demonstrate, the commonly used fluctuation-based approach also exhibits strong concentration dependence in practice, particularly for fluorescent proteins prone to weak self-association. Even modest changes in sample concentration can elevate the apparent brightness due to transient or low-affinity oligomerization, leading to overestimates of the monomeric reference. Therefore, our findings support a multipronged strategy for brightness calibration.

In addition to addressing monomeric brightness calibration challenges in isolation, this work also contributes to a broader class of fluorescence techniques where accurate monomeric brightness measurements are essential. One such example is intensity fluctuation and resonance energy transfer (iFRET) [36]. In iFRET, donor and acceptor intensity fluctuations within a small subregion in the sample are analyzed simultaneously alongside the mean donor and acceptor intensities (which allow computation of the FRET efficiency occurring between the two) within said region, to extract information about the size, spatial organization, and distribution of molecular complexes in living cells. By integrating the complementary information provided by variance- and mean-based readouts from the same dataset, iFRET reduces ambiguity and increases interpretive power.

A similar philosophy underlies the present calibration strategy: although average-intensity and fluctuation-based estimates of molecular brightness each have distinct strengths and limitations, their joint application provides cross-validating constraints that improve the robustness of both. In this light, it is fitting that the same principle of methodological complementarity not only enhances the accuracy of monomeric brightness calibration itself, but also directly benefits iFRET workflows. In particular, accurately calibrated intensity-based brightness enables more rigorous fluctuation analysis in iFRET, where assumptions about monomeric brightness critically influence derived complex sizes and populations. Thus, the methods developed here serve not only to benchmark fluorophore brightness, but also to enhance the precision and reliability of iFRET and other fluorescence fluctuation spectroscopy techniques.

## 2. Results

### 2.1. Estimating the Effective Excitation Volume Using Fluorescent Nanobeads

In this study, we used a custom-built two-photon microscope, described previously [37,38], which combines a pulsed Ti:Sapphire laser with a tunable center wavelength, a spatial light modulator that shapes the excitation beam into multiple focused spots, and an EMCCD camera for sensitive fluorescence detection. This setup enables simultaneous measurements at multiple locations, making it well-suited for high-throughput brightness analysis of proteins in solution.

To determine the effective excitation volume required for intensity-based molecular brightness calculations, we used sub-diffraction fluorescent nanobeads embedded in a transparent hydrogel matrix (see Section 4.1.1). These beads serve as point-like emitters that allow precise mapping of the microscope’s focal volume. By scanning the beads laterally and axially under excitation conditions matched to those used in protein measurements, we extracted key optical parameters of the beam: the lateral beam waist ($w_{0}$) and the axial Rayleigh length ($z_{R}$).

To map the beam’s lateral profile, we measured the fluorescence intensity across individual nanobeads in the focal plane. Because fluorescence from two-photon excitation depends on the square of the beam intensity, we analyzed the square root of the signal to better reflect the beam’s actual shape. The resulting profiles were fit to a Gaussian curve, and the beam width was characterized using the 1/e^2^ radius, a standard measure of beam spread. A similar approach was used axially by scanning through the bead in small steps along the z-direction and fitting the vertical intensity profile. See Section 4.2.2 for more details on this process.

**Figure 1:**
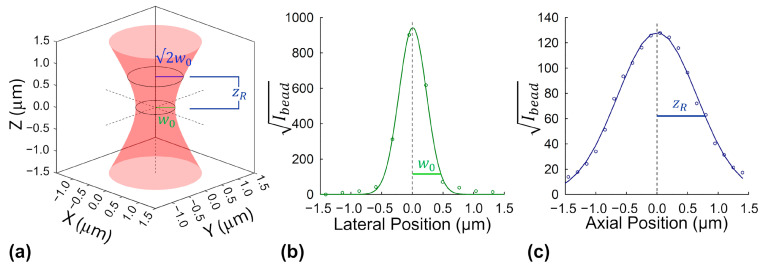
Representative nanobead fluorescence profiles used for lateral, axial, and volumetric excitation beam characterization. (**a**) Three-dimensional isosurface rendering (semi-transparent red mesh) of the modeled two-photon excitation volume. The surface represents the 1/e^2^ contour of the relative intensity, where intensity falls to 1/e^2^ of the on-axis peak at each axial position. Black dashed lines denote the principal axes, and solid rings at z=0 and $z=z_{R}$ denote the transverse 1/e^2^ beam radii $w_{0}$ and $w\left(z_{R}\right)$, shown with green and blue radial lines, respectively. (**b**) The square root of the measured fluorescence intensity from a single nanobead in the focal plane was plotted against pixel position along a line through the bead. Pixel positions were converted to physical distance using a 272 nm/pixel calibration. The resulting profile was fit with a 1D Gaussian function, yielding a 1/e^2^ width of 448.5 nm for this example. The widths from 16 different beads were averaged and corrected for the finite size of the beads (see Equation (4) in Section 4.2.2), yielding an average beam waist of $w_{0}=424\pm 40\;nm$. (**c**) Axial fluorescence profile of a single nanobead. The square root of the integrated fluorescence (5 × 5 pixel area) from a single bead, recorded across a z-stack at 150 nm intervals, was plotted versus axial position and fit with a Gaussian. Profiles from 21 such beads were fit individually to extract their 1/e^2^ widths, which were then averaged and converted to a Rayleigh length of $z_{R}=703\pm 71\;nm$. The values of $w_{0}$ and $z_{R}$ were used along with Equations (6)–(8) to compute an effective excitation volume of $V_{PSF}=0.28\;fL$. In panels b and c, the square root transformation accounts for the quadratic dependence of two-photon excitation on beam intensity.

The final averaged $w_{0}$ and $z_{R}$ parameters were used to calculate the effective excitation volume, as described in Equations (5)–(8). Although the measured Rayleigh range slightly exceeded (by ~20%) the theoretically predicted value for a diffraction-limited Gaussian beam with a width of $w_{0}$, the empirical value was used for all downstream volume calculations, yielding an effective excitation volume of $V_{PSF}=0.28\;fL$. This calibrated volume was subsequently used in all intensity-based monomeric molecular brightness estimations throughout the study. Representative bead profiles in both lateral and axial directions are shown in Figure 1.

### 2.2. Framework for Comparing Fluctuation-Based and Intensity-Based Brightness Estimation

To assess the robustness and consistency of monomeric molecular brightness estimation across different experimental conditions, we employed a dual-analysis strategy that compares two approaches: (1) fluctuation-based analysis, which estimates brightness from temporal intensity fluctuations in fluorescence traces, and (2) intensity-based calibration, which derives brightness from the average fluorescence intensity, known fluorophore concentration, and a calibrated effective observation volume. This framework enables direct comparison between the traditional fluctuation-derived approach of determining monomeric brightness and the proposed average intensity-based method, particularly in systems where aggregation may distort fluctuation-derived estimates. The same data acquisition and analysis pipeline, described below, was applied to a variety of fluorophore samples under different preparation conditions, as detailed in subsequent sections.

Fluorescence measurements of fluorophore solutions were carried out using the custom two-photon microscope described in Section 4.2.1. The excitation beam was patterned into a fixed 6 × 1 array, illuminating six distinct spots within the sample chamber simultaneously. With the beamlets held stationary during acquisition, 10,000 consecutive frames were captured at 100 µsec exposure time per frame, producing six independent fluorescence intensity traces—one for each beamlet. This process was repeated across multiple sample positions to improve statistical sampling. The resulting traces were then analyzed using both fluctuation- and intensity-based approaches to estimate the monomeric molecular brightness.

In the fluctuation-based approach, an effective molecular brightness value, $\varepsilon_{eff}$, was derived from each intensity trace based on the variance, $\sigma^{2}$, and mean, ⟨$I_{s}$⟩, of the intensity distribution according to the following [7,11]:

(1) $$\varepsilon_{eff}=\frac{\sigma^{2}-\sigma_{D}^{2}}{\gamma \langle I_{s}\rangle }$$

**Figure 2:**
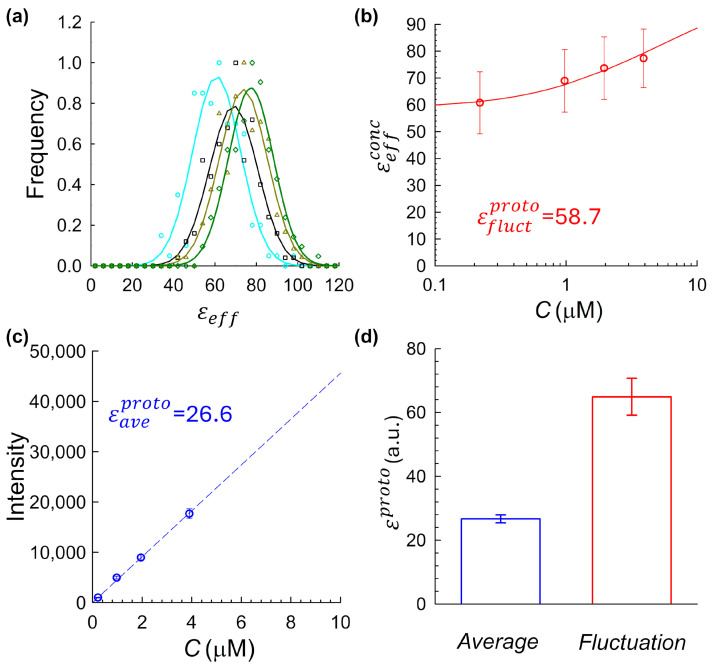
Comparison of fluctuation-derived and average-intensity-derived molecular brightness for mCitrine under uninhibited-aggregation conditions. (**a**) Histograms of molecular brightness values at multiple concentrations, showing a rightward shift with increasing concentration, consistent with concentration-dependent oligomerization. Each was fit to a Gaussian to extract the mean effective brightness for that concentration, $\varepsilon_{eff}^{conc}$. (**b**) Fluctuation-derived brightness vs. fluorophore concentration. Each data point corresponds to the $\varepsilon_{eff}^{conc}$ value obtained by Gaussian fitting of the corresponding histogram in panel (**a**) at the respective fluorophore concentration. The data were fit using a monomer–dimer equilibrium model (Equation (2)) to extrapolate the monomeric brightness. (**c**) Average fluorescence intensity vs. fluorophore concentration. The linear fit slope Θ was used to estimate intensity-based monomeric molecular brightness, $\varepsilon_{ave}^{proto}$, via Equation (3). (**d**) Summary comparison across three independently prepared mCitrine samples, each processed using the same analysis pipeline shown in panels (**a**–**c**). Each bar represents the mean monomeric brightness extracted via fluctuation-based (right) and average-intensity-based (left) methods. Error bars indicate the standard deviation across N=3 independent preparations. Fluctuation-derived estimates were consistently higher than those obtained from the average-intensity method, underscoring their sensitivity to aggregation artifacts.

Here $\sigma_{D}^{2}$ represents the variance due to detector noise (determined from calibration measurements [11]), and γ is a geometric shape factor related to the laser point-spread function (PSF) and acquisition volume [14]. The resulting $\varepsilon_{eff}$ values from all traces at a given concentration were compiled into a histogram (Figure 2a). These histograms were fit to Gaussian functions, and the mean of each fit, denoted as $\varepsilon_{eff}^{conc}$, was used as the representative brightness value for the corresponding fluorophore concentration.

Figure 2b shows $\varepsilon_{eff}^{conc}$, as a function of the measured fluorophore concentration. Fluorophore concentrations were independently verified by UV-Vis absorption spectroscopy. Across all tested samples, a consistent upward trend in brightness with increasing concentration was observed, indicating a degree of fluorophore self-association or aggregation, a pattern that varied in magnitude depending on the fluorophore and sample preparation conditions (as further detailed in Section 2.3, Section 2.4 and Section 2.5 below). To quantify this effect and extrapolate the monomeric baseline brightness computed via the fluctuation approach, $\varepsilon_{fluct}^{proto}$, we fit the fluctuation-derived brightness data using a model based on the Law of Mass Action, assuming a simple monomer–dimer equilibrium [11]. In this framework, the total effective brightness at a given fluorophore concentration C is expressed as

(2) $$\varepsilon_{theo}^{conc}=\;\varepsilon_{fluct}^{proto}\left(2-\frac{K_{d}}{4C}\left(\sqrt{1+\frac{8C}{K_{d}}}-1\right)\right)$$

where $\varepsilon_{fluct}^{proto}$ is the monomeric baseline brightness, $K_{d}$ is the dissociation constant, and C is the total concentration of fluorophores of interest. This model was fitted to the experimental data using nonlinear regression, allowing both $\varepsilon_{fluct}^{proto}$ and $K_{d}$ to vary as free parameters. The fitted value of $\varepsilon_{fluct}^{proto}$ provides the intrinsic monomeric brightness inferred from the fluctuation approach.

In the average intensity-based approach, we analyzed the same intensity traces described above using an average intensity vs. concentration method. For each fluorophore concentration, the mean fluorescence signal from every trace was computed and averaged across replicates at each concentration. The resulting plot of average signal versus concentration (Figure 2c) was fitted with a linear regression., as shown in Figure 2c [31,32]. The resulting slope of this fitted line, Θ, represents the detected fluorescence intensity per unit concentration (in digital units per µM). This value was then converted to monomeric molecular brightness computed via the intensity-based method, $\varepsilon_{ave}^{proto}$, by dividing by the empirically measured observation volume:

(3) $$\varepsilon_{ave}^{proto}=\frac{\Theta }{\nu_{PSF}}$$

where the calibrated volume $V_{PSF}$ was derived from nanobead-based beam profiling (Section 2.1).

To summarize the notation used throughout this work to describe molecular brightness estimates: we use *ε* to denote the molecular brightness of a fluorophore. The term $\varepsilon_{eff}$ refers to the effective brightness measured for a given sample, which could reflect a heterogeneous population of monomers, dimers, and higher-order species. Repeat measurements across different locations within the same sample yield a distribution of $\varepsilon_{eff}$ values; these are compiled into histograms and fit with Gaussians, and the resulting means are denoted $\varepsilon_{eff}^{conc}$, which represents the mean $\varepsilon_{eff}$ for a sample for a single concentration. In contrast, a superscript “proto” ($\varepsilon^{proto}$) indicates a reference monomeric brightness value, measured under conditions chosen to minimize self-association. Subscripts “fluct” ($\varepsilon_{fluct}^{proto}$) and “ave” ($\varepsilon_{ave}^{proto}$) specify the method used to determine monomeric brightness, either via fluctuation-based analysis or average-intensity-based calibration, respectively.

To evaluate reproducibility and agreement between the two molecular brightness estimation strategies, this dual-analysis workflow was repeated across multiple independent experimental replicates. For each replicate, brightness was calculated both from fluorescence fluctuations and from the average intensity–based method described above. Subsequent sections explore how this comparison evolves across fluorescent proteins with varied aggregation tendencies. These include mCitrine under varying sample preparation conditions and JF_525_–HaloTag ligand as a minimally aggregating control. In each case, both brightness estimation methods were applied using the same data acquisition framework, enabling direct, quantitative comparison.

### 2.3. mCitrine Under Aggregation-Prone Conditions Reveals Divergence Between Brightness Methods

The first complete dual-method analysis was performed using an mCitrine sample that, unintentionally, omitted several of the handling steps later found to be important for reducing aggregation and isolating monomeric species (see Section 4.1.2). Specifically, the sample was not flash-frozen, was not originally eluted into a dithiothreitol (DTT)-containing buffer, and although it was processed by fast protein liquid chromatography (FPLC), the collected fraction spanned a broad elution window that likely included higher-order species. This dataset was initially collected during early validation of the intensity-based brightness calibration procedure and was expected to represent a reliable monomeric control, as it used mCitrine containing the A206K monomerizing mutation. However, the sample was later found to be aggregation-prone, providing an unexpected yet illustrative test case for comparing the two analysis methods and demonstrating that protein aggregation could strongly distort fluctuation-based monomeric brightness estimates.

Using the fluorescence acquisition and analysis pipeline described in Section 2.2, we computed monomeric molecular brightness values using both fluctuation- and -intensity-based methods. For the fluctuation approach, histograms of $\varepsilon_{eff}$ values were generated for each concentration (Figure 2a) and fit with Gaussian functions. The resulting fitted means, $\varepsilon_{eff}^{conc}$, were plotted as a function of fluorophore concentration (Figure 2b), revealing a clear upward trend across the tested range. This increase strongly suggests fluorophore self-association. Importantly, because $\varepsilon_{eff}^{conc}$ varies with concentration, no single concentration provides an unambiguous monomeric baseline, underscoring a fundamental challenge in using this approach alone. To estimate a true monomeric brightness value, we fit the data shown in Figure 2b using a monomer–dimer equilibrium model based on the Law of Mass Action (Equation (2)), yielding a monomeric baseline of $\varepsilon_{fluct}^{proto}=58.7$. However, applying the average-intensity-based method to the same data yielded a markedly lower monomeric brightness value of $\varepsilon_{ave}^{proto}=26.6\;$ (Figure 2c). To assess reproducibility, the full dual-method analysis was repeated across three independently prepared mCitrine samples, using the same aggregation-prone protocol. As shown in Figure 2d, fluctuation-derived estimates of monomeric brightness were consistently higher than those obtained using the intensity-based method. This substantial discrepancy raised a central question: was one method failing, or were the two methods fundamentally sensitive to different aggregation-related artifacts?

To explore discrepancy between methods further, we subjected the same mCitrine preparation to FPLC fractionation and collected two narrow elution peaks corresponding to distinct apparent molecular sizes (Supplementary Figure S1). Brightness measurements were then repeated on both fractions under matched concentration conditions. In the fluctuation-derived brightness, the earlier FPLC peak (corresponding to a larger-size) showed a markedly higher $\varepsilon_{eff}^{conc}$ value. In contrast, the average-intensity-derived brightness remained essentially unchanged across both fractions (see Supplementary Figure S1). This result pointed to the presence of higher-order oligomers in the larger-size peak, which inflate fluctuation-derived brightness estimates due to their disproportionate signal contribution, yet contribute proportionally to total fluorescence intensity at fixed concentration.

These results provide initial evidence that aggregation can substantially distort fluctuation-based brightness estimation, whereas the average-intensity calibration approach remains comparatively stable, even in the presence of molecular heterogeneity. This contrast suggests that intensity-based methods, when paired with accurate concentration and volume calibration, may offer a more consistent path to monomeric brightness estimation in aggregation-prone systems. Notably, this holds true even for fluorescent proteins engineered with monomerization mutations (e.g., A206K), indicating that standard constructs alone may not be sufficient to eliminate self-association effects. In the following section, we test this hypothesis under more rigorously controlled preparation conditions using the same fluorophore.

### 2.4. mCitrine Under Controlled Preparation Conditions Yields Improved Agreement Between Brightness Methods

**Figure 3:**
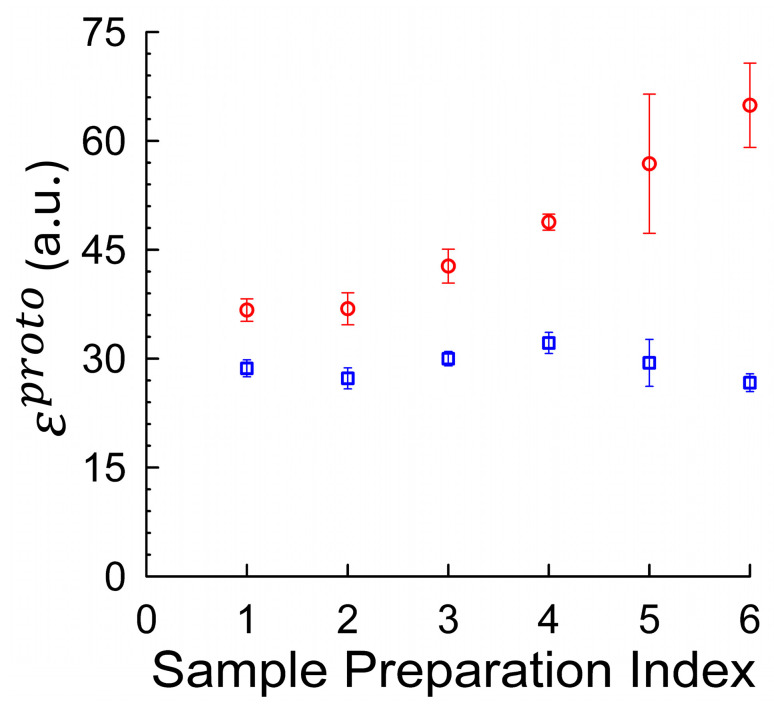
Sensitivity of molecular brightness estimates to sample preparation conditions in mCitrine. Monomeric brightness was computed using both fluctuation-based (red circles) and intensity-based (blue squares) methods across six different sample preparation protocols (Index 1–6) summarized in Table 1; see Section 4.1.2 for detailed conditions. Error bars represent standard deviation across replicate measurements; the number of replicates for each condition is provided in Table 1. Each index represents a unique combination of purification and storage conditions, including the use or omission of DTT, PEG cryoprotectant, FPLC fractionation, and flash-freezing. The fluctuation-derived estimates (red circles) show a strong dependence on preparation conditions, increasing from 36.7 to 64.9 across the six indices, indicative of sample-dependent aggregation. In contrast, average-intensity-derived estimates (blue squares) remain comparatively stable, varying only modestly across conditions. This divergence highlights the susceptibility of fluctuation-based brightness methods to aggregation artifacts, and the robustness of the intensity–volume–concentration calibration approach.

To systematically explore how sample handling influences the agreement between brightness estimation methods, we analyzed six distinct mCitrine preparation protocols, indexed 1–6 (See Table 1). These protocols varied in key factors, such as FPLC elution buffer composition (±1 mM DTT), storage condition (fresh vs. flash-frozen), inclusion of 1% polyethylene glycol (PEG) as a cryoprotectant during freezing, and the extent of size-based fractionation via FPLC (Supplementary Figure S1). 10 mM 4-(2-hydroxyethyl)-1-piperazineethanesulfonic acid (HEPES) at pH 7.2 was used as the standard buffer throughout to ensure stable, near-physiological pH throughout sample handling and measurement. DTT (1 mM) was included in some preparations to maintain reducing conditions and prevent disulfide-mediated aggregation. PEG (1% *w*/*v*) was added as a cryoprotectant in selected samples to reduce aggregation induced during flash freezing. For each indexed condition, monomeric molecular brightness was computed using both fluctuation-based ($\varepsilon_{fluct}^{proto}$) and average-intensity-based approaches ($\varepsilon_{ave}^{proto}$). Figure 3 summarizes the results across all six protocols. The fluctuation-derived brightness values (red circles) showed pronounced variability across conditions, ranging from 36.7 to 64.9 units per molecule. In contrast, the average-intensity-derived brightness (blue squares) remained relatively stable, with values clustered between 27 and 32 units. This divergence underscores the sensitivity of fluctuation analysis to even modest aggregation levels, while highlighting the comparative robustness of the intensity-based calibration method.

To further investigate conditions that yield the best agreement between brightness estimation methods, we examined the sample indexed by 2, which was eluted into 1 mM DTT during high-performance liquid chromatography (HPLC) purification and flash-frozen in the presence of 1% PEG (*w*/*v*) as a cryoprotectant to reduce oxidation and aggregation. To estimate the sensitivity of the fluctuation-based method to even minor handling artifacts, we compared these results to Index 3, which differed only in the omission of PEG during freezing. Despite identical buffer conditions, this small change produced a marked elevation in fluctuation-derived brightness, consistent with freezing-induced aggregation (see Supplementary Figure S2).

**Figure 4:**
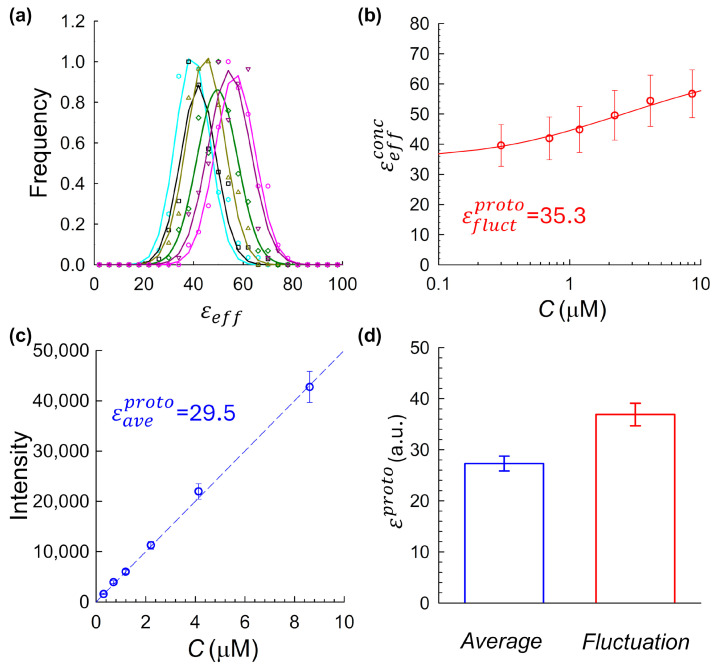
Comparison of fluctuation-derived and average-intensity-derived molecular brightness for mCitrine under controlled preparation conditions (Index 2). (**a**) Histograms of molecular brightness values at multiple concentrations, showing a rightward shift with increasing concentration, consistent with concentration-dependent oligomerization. Each was fit to a Gaussian to extract the mean effective brightness for that concentration, $\varepsilon_{eff}^{conc}$. (**b**) Fluctuation-derived brightness values plotted as a function of fluorophore concentration. Each data point corresponds to the $\varepsilon_{eff}^{conc}$ value obtained by Gaussian fitting of the corresponding histogram in panel (**a**) at the respective fluorophore concentration. An upward trend suggests weak concentration-dependent self-association, even under improved sample preparation conditions. (**c**) Average fluorescence intensity plotted versus fluorophore concentration. The linear fit slope Θ was used to estimate molecular brightness via Equation (3). (**d**) Summary comparison across five independent mCitrine preparations. Each bar represents the mean monomeric brightness extracted via fluctuation-based (right) and average-intensity-based (left) methods, with error bars denoting standard deviation across N=5 independent replicates.

Under these more controlled conditions, five independent mCitrine preparations were each measured across a range of concentrations to enable full brightness calibration using both fluctuation-based and intensity-based approaches. Figure 4 shows results from one of these five independent experiments, laid out using the same panel structure as in Figure 2. Histograms of fluctuation-derived brightness values computed from intensity traces across a range of concentrations were fit by Gaussian functions to extract the mean brightness for each concentration sample (panel a). These values, plotted against concentration in panel b, revealed a clear upward trend, suggestive of weak concentration-dependent self-association, even under these controlled conditions. The slope of the intensity–concentration plot yielded a molecular brightness of 29.5. Panel d summarizes the results across all five replicates of samples prepared according to index 2, shown as a bar chart with means and standard deviations. On average, fluctuation-based analysis yielded an average value of $\varepsilon_{fluct}^{proto}=36.9\pm 2.2$, which is significantly different than the value of $\varepsilon_{ave}^{proto}=27.3\pm 1.5$ from the intensity-based method. Importantly, reproducibility of mCitrine brightness measurements required careful handling. Brightness estimates were only compiled from samples that were either (a) freshly purified and immediately measured (see Section 4.1.2), or (b) flash-frozen in the presence of 1% PEG as a cryoprotectant to minimize aggregation.

Together, these findings show that while careful sample handling improves agreement between the two brightness estimation methods, a discrepancy still remains. This persistent gap underscores the need for further validation of the intensity-based calibration approach using an alternative fluorophore with minimal aggregation propensity, which is discussed next.

### 2.5. JF_525_–HaloTag Ligand as a Minimally Aggregating Benchmark System

To further compare the accuracy of the two molecular brightness-determination approaches, we used protein–fluorophore system that is theoretically less prone to aggregation: the Janelia Fluor 525 (JF_525_)–HaloTag conjugate. HaloTag is widely used in quantitative fluorescence studies and, in multiple experimental contexts, has been shown to exhibit reduced self-association compared to aggregation-prone fluorescent proteins [34,35]. In these studies, both HaloTag and fluorescent proteins were fused to the same protein of interest, and the aggregation behavior of the fusion constructs was compared. HaloTag fusions preserved the dynamic or phase behavior of the target proteins more faithfully, suggesting a lower propensity for self-association in this context. However, direct, quantitative comparisons to mCitrine and related variants remain limited in the literature. It was for this reason that we conducted pKa-based electrostatic modeling using the Rosetta-pH algorithm to assess the aggregation propensity of the HaloTag conjugate under physiological pH conditions [39,40]. The Rosetta-pH modeling estimated a net charge of −33 for HaloTag at pH 7.2, expressed in units of elementary charge (e). This value reflects the total protonation state of all ionizable residues, as predicted from the protein structure and local environment at the specified pH. Notably, previous studies have shown that increased negative surface charge correlates strongly with improved protein solubility and reduced aggregation tendency [41]. Therefore, the substantial negative charge obtained from Rosetta simulations supports our use of HaloTag as a minimally aggregating reference system. For comparison, we also performed Rosetta-pH modeling on Citrine, which yielded a net charge of −3e at pH 7.2. Although the structure lacked the A206K mutation present in our mCitrine construct, the large discrepancy in net electrostatic charge between the two proteins, HaloTag (−33e) vs. Citrine (−3e), supports the reduced aggregation propensity of HaloTag and its suitability as a solubility reference. Combined with JF_525_, a bright, rhodamine dye developed by the Lavis Lab at Janelia Research Campus [33], the system serves as a useful benchmark for evaluating brightness estimation methods where aggregation is expected to be minimal.

**Figure 5:**
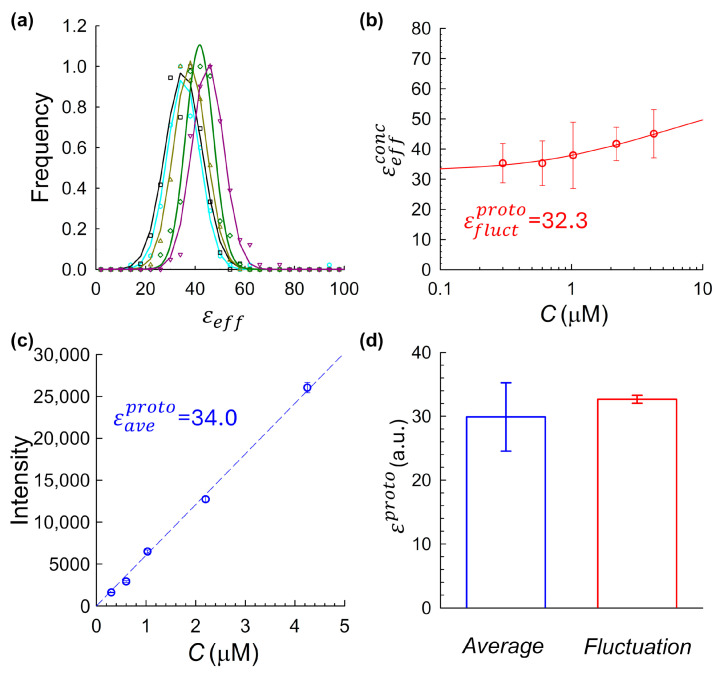
Comparison of fluctuation-derived and average-intensity-derived molecular brightness for JF_525_–HaloTag conjugates. (**a**) Histograms of molecular brightness values at multiple concentrations, showing a rightward shift with increasing concentration, consistent with concentration-dependent oligomerization. Each was fit to a Gaussian to extract the mean effective brightness for that concentration, $\varepsilon_{eff}^{conc}$. (**b**) Fluctuation-derived brightness values plotted as a function of fluorophore concentration. Each data point corresponds to the $\varepsilon_{eff}^{conc}$ value obtained by Gaussian fitting of the corresponding histogram in panel (**a**) at the respective fluorophore concentration. A mild upward trend is observed, consistent with weak self-association. The data were fit using a monomer–dimer equilibrium model (Equation (2)) to extract the monomeric baseline brightness. (**c**) Average fluorescence intensity plotted versus fluorophore concentration. The slope Θ of the linear fit was used to estimate molecular brightness via Equation (3). (**d**) Summary comparison across four independent replicates. Each bar represents the mean monomeric brightness extracted from either the fluctuation-based or intensity-based method, with error bars denoting standard deviation across N=4 independent replicates. The close agreement between the two methods supports the accuracy and reproducibility of the intensity-based approach.

To experimentally evaluate the system, we applied both fluctuation-based and intensity-based brightness estimation methods to JF_525_–HaloTag conjugates across a range of concentrations, as summarized in Figure 5. The fluctuation-derived brightness showed only a modest concentration-dependent increase (Figure 5b), consistent with weak self-association. Although smaller in magnitude than for mCitrine, this trend suggests a degree of residual aggregation at higher concentrations. These effects were measurable but minor, reinforcing the suitability of JF_525_–HaloTag as a comparative benchmark system. The intensity-based method yielded a closely matching monomeric brightness estimate of (Figure 5c). To evaluate the reproducibility of the agreement between the two molecular brightness estimation strategies, we repeated the dual analysis described above across four independent experimental replicates, each using freshly prepared JF_525_–HaloTag samples. Across these replicates, the fluctuation-based method produced a mean brightness of $\varepsilon_{fluct}^{proto}=32.7\pm 0.6$, while the average-intensity-based method yielded $\varepsilon_{ave}^{proto}=29.9\;\pm 5.3$ (Figure 5d). Although the results are in close agreement, a modest residual discrepancy persists, likely reflecting small variations in the intensity-based estimate, which is sensitive to factors such as fluorophore extinction coefficient in the measurement buffer, and pH mismatch between cuvette and imaging chambers. Still, the overall convergence strongly supports the average-intensity calibration method as a reliable route to determining molecular brightness, particularly when used in tandem with accurate concentration and volume characterization.

## 3. Discussion

Quantitative fluorescence fluctuation spectroscopy (FFS), including applications such as iFRET [36] and SpiDA [9,10], relies heavily on accurate estimates of the monomeric molecular brightness to infer stoichiometry and oligomeric states of the proteins of interest [42,43]. However, the results presented here underscore the inherent difficulty of establishing reliable monomeric brightness standards using fluctuation-based approaches alone, particularly for fluorophores prone to self-association. Even constructs engineered to be monomeric, such as mCitrine with the A206K mutation, exhibited strong concentration-dependent brightness increases under standard experimental conditions. These artifacts can arise from transient interactions, low-level aggregation, or incomplete separation of oligomeric species during purification. A key challenge is that, in such cases, the inferred brightness of the “monomeric control” is not constant, but varies systematically with fluorophore concentration, compromising its utility as a baseline standard because the value extracted from calibration measurements depends on the concentration of the samples undergoing measurement.

This study highlights a complementary strategy for estimating monomeric molecular brightness based on measured fluorescence intensity, independently verified fluorophore concentration, and an empirically determined size for the observation volume. In multiple experimental contexts, this average-intensity method demonstrated greater stability than traditional fluctuation-based estimates, particularly in scenarios where self-association or sample heterogeneity introduced non-monomeric species. In the well-controlled JF_525_–HaloTag system, both methods yielded closely matching results, validating the method’s accuracy under aggregation-minimized conditions. In contrast, for more aggregation-prone systems such as mCitrine, the intensity-based method was less sensitive to sample handling variability and produced more consistent brightness estimates across replicates, even in preparations where the fluctuation-derived values were elevated. Thus, the divergence between the two methods by itself carries diagnostic value. A widening gap, especially when the intensity-based estimate remains stable, can signal oligomeric contamination or ongoing molecular interactions.

At first glance, the average-intensity-based approach may appear limited by its reliance on externally measured quantities: namely, fluorophore concentration and the effective observation volume. However, as this study demonstrates, the commonly used fluctuation-based approach is not exempt from similar dependencies to fluorophore concentration. Particularly for fluorescent proteins that exhibit weak or transient self-association, even small variations in concentration can produce substantial changes in apparent brightness, leading to inflated monomeric baselines. In such cases, the fluctuation-derived brightness baseline is itself concentration-dependent, undermining its role as a fixed calibration point. While accurate determination of concentration is relatively straightforward in well-controlled solution- experiments, such calibration is more difficult in spatially heterogeneous environments, such as membranes or cellular organelles [44,45]. Moreover, even when concentration is well characterized, extracting monomeric brightness from brightness-versus-concentration data requires assumptions about the underlying interaction model, for example, fitting to a simple monomer–dimer equilibrium may not capture systems with higher-order or multi-state associations, potentially confounding interpretation.

While more elaborate binding models might improve the fit to these datasets—incorporating factors such as multi-site interactions, higher-order oligomerization, or dark fluorophore fractions—to better fit the concentration-dependent brightness curves, such approaches introduce significant modeling complexity. In the case of mCitrine, variability across preparations suggests that distinct interaction mechanisms or aggregation pathways may be at play, making it difficult to apply a single reliable model. While such strategies may be appropriate for targeted mechanistic studies, the dimerization model is used in this work as a tool for determining the oligomer brightness by simple extrapolation of the theoretical fit to low concentrations of proteins. Rather than investing effort into explaining the behavior of a “monomeric control,” we show that a more stable, orthogonal calibration method frees researchers to focus their modeling on systems where the biology is meaningful, such as ligand-induced receptor oligomerization in membrane environments [11,12,23,46].

Despite its robustness, the intensity-based method relies on several important assumptions. First, it depends on accurate fluorophore concentration measurements, typically obtained via absorbance spectroscopy using extinction coefficients that may vary with buffer composition, pH, or labeling environment [47,48]. Second, fluorescence yield can be modulated by the local chemical environment, meaning that calibration performed in bulk solution may not accurately reflect intracellular behavior. Third, the method assumes linear excitation conditions, where fluorescence intensity scales proportionally with excitation power. As shown by Nagy, Wu, and Berland [15,30], this linearity holds in the low-power regime but breaks down at higher excitation intensities, where photon absorption approaches or exceeds the spontaneous emission rate. This leads to excitation saturation, distorting the PSF shape and invalidating volume models based on Gaussian optics. To avoid such artifacts, all experiments in this study were performed under carefully validated linear conditions. However, when applying the method to other systems, users should independently verify that excitation remains in the linear regime to ensure accurate quantification.

Finally, the intensity-based approach assumes a well-characterized and stable observation volume, $V_{PSF}$, derived from the squared point-spread function (PSF^2^). In this study, the volume was estimated using nanobead-based 3D PSF profiling, constructed by stepping the objective in fixed axial increments (150 nm). However, in certain instances, the actual step size may deviate from the commanded value due to things such as mechanical backlash, thermal expansion, or positioning errors inherent to motorized systems. These deviations can distort the integrated PSF^2^ profile and introduce systematic over- or underestimation of the observation volume, even within a single acquisition, particularly during z-stack sequences. While averaging across multiple beads helps reduce variability, it cannot fully correct for such systematic distortions. Future implementations could benefit from hardware-based axial stabilization, such as FREVR (Focus Readjustment for Enhanced Vertical Resolution) [49], which uses interferometric feedback to maintain sub-nanometer z-positioning throughout acquisition.

It is also important to note that all measurements in this study were acquired on a custom-built two-photon micro-spectroscope, as described in Section 4.2.1 and in previous work [38]. The absolute excitation volume reported here is therefore instrument specific. Implementing the intensity-based calibration strategy on other microscopes will require analogous characterization of the point-spread function and observation volume on each platform, together with verification that excitation remains in the linear regime.

While the primary focus of this work was to establish a robust calibration strategy for monomeric molecular brightness, it has not escaped our attention that the slope-based method described here, when used in conjunction with fluctuation-derived brightness estimates, can be extended to determine equilibrium dissociation constants by analyzing changes in apparent oligomeric state across a range of concentrations, particularly under conditions where molecular interactions are preserved (*K_D_*). Extracting reliable *K_D_* values in this context requires a carefully controlled system with known labeling stoichiometry, non-perturbative measurement conditions, and constructs that retain reversible binding properties over a measurable concentration range. In our present manuscript we have pursued the opposite goal and therefore chose samples and experimental conditions that reduce oligomerization. However, we are actively pursuing this line of approach in a parallel study using mEGFP-derived dimers and higher-order oligomers to evaluate this combined strategy for benchmarking *K_D_* estimates under physiological and non-inhibitory conditions. These findings will be described in future publications.

The findings presented here underscore the need for rigorous, validated approaches to monomeric brightness calibration in FFS. While fluctuation-based analyses using “monomeric controls” are standard practice, such controls are vulnerable to low-level aggregation that can distort monomeric brightness estimates. This study demonstrates that calculating molecular brightness from average fluorescence intensity, known fluorophore concentration, and a carefully calibrated observation volume offers a reliable and complementary alternative, one that is less susceptible to aggregation artifacts and preparation variability. Neither method is universally robust in isolation: both carry assumptions and limitations. Instead, a dual-calibration strategy that leverages both approaches provides cross-validation, improves detection of aggregation-related distortions, and strengthens confidence in monomeric baselines. While JF_525_–HaloTag served as a validation system here, monomeric brightness calibration is ideally performed using the same fluorophore employed in the experiment, to capture condition-specific behavior. In aggregation-prone constructs such as mCitrine, the intensity-based method yielded more consistent brightness values across replicates, even when fluctuation-derived estimates were distorted by non-monomeric species. Used in tandem, intensity- and fluctuation-based approaches provide complementary insights, internal consistency checks, and enhanced confidence in interpreting molecular stoichiometry in complex biological systems.

## 4. Materials and Methods

### 4.1. Sample Preparation

#### 4.1.1. Nanobead Preparation for Excitation Volume Calibration

To estimate the effective excitation volume of the laser focal volume, we used 170 nm fluorescent nanobeads (PS-Speck; Invitrogen, Carlsbad, CA, USA; cat. no. P7220) embedded in Cygel™ (BioStatus Unlimited, Shepshed, UK), a thermoreversible hydrogel. Beads were prepared by mixing 4 µL of the bead suspension into 200 µL of Cygel, which was then deposited into the wells of BSA-passivated imaging chambers as described in Section 4.1.4. After gelation at room temperature, the samples were mounted on the microscope for imaging.

#### 4.1.2. mCitrine Solution Preparation

The mCitrine constructs used in fluorescent protein solution experiments were cloned into a modified pQE-80L expression vector containing a (His)_6_tag at the N-terminus and a (Cys)_2_ at the C-terminus. Expression plasmids were transformed into *E. coli* C41(DE3) cells (Sigma-Aldrich, Burlington, MA, USA), which were grown at 37 °C in the presence of carbenicillin to an OD^600^ of 0.7. Protein expression was induced with 1 mM IPTG and carried out overnight at 25 °C. Cells were harvested by centrifugation, resuspended in sodium phosphate buffer (pH 7.0, 300 mM NaCl; referred to as EW buffer) and lysed by lysozyme treatment followed by sonication. The soluble fraction was applied to a Ni-NTA affinity column (GE Healthcare, Waukesha, WI, USA), washed with EW buffer containing 7.5% imidazole, and eluted in EW buffer with 250 mM imidazole. Eluted protein was immediately subjected to size-exclusion chromatography (Superdex 200 10/300 GL, GE Healthcare), and collected in HEPES buffer (10 mM HEPES, 150 mM NaCl, pH 7.2). The highest concentration fractions, determined based on absorbance at 515 nm, were saved.

Following purification, protein samples were divided into aliquots and subjected to different downstream treatment conditions to assess the effects of storage and buffer composition. Specifically, some samples were collected in HEPES buffer with 1 mM DTT, while others lacked DTT. Aliquots were either measured immediately (fresh) or flash-frozen and stored at −80 °C. Among the frozen samples, some were prepared in the presence of 1% PEG (*w*/*v*) as a cryoprotectant, while others were frozen without PEG. These six sample preparation conditions were indexed for clarity and are summarized in Table 1. For each measurement set, an mCitrine solution prepared under one of the six treatment conditions was first quantified by absorbance at 516 nm, using an extinction coefficient of 94,000 M^−1^cm^−1^ [50]. From this stock, 4–6 distinct concentrations were prepared by direct dilution and used for subsequent measurements.

#### 4.1.3. HaloTag Ligand Preparation

JF_525_–HaloTag ligand was obtained in powder form from the Janelia Research Campus (Ashburn, VA, USA), diluted in dimethyl sulfoxide (DMSO) to a concentration of 150 µM, and stored in aliquots at −80 °C until use. Prior to each experiment, a single aliquot was thawed and mixed with purified HaloTag–SpyTag protein at a slight molar excess of protein (0.8:1 fluorophore:protein ratio) in buffer containing 50 mM HEPES, 150 mM NaCl, 1 mM DTT at pH 7.2 to create JF_525_–HaloTag conjugates. The reaction mixture was then diluted tenfold with the same buffer and incubated on ice for at least one hour to ensure the complete dye-binding. Following incubation, the mixture was centrifuged at 4 °C using Amicon ultra centrifugal filters with a molecular weight cut-off (MWCO) of 10 kDa (Sigma-Aldrich, Burlington, MA, USA) to remove unbound fluorogenic JF_525_–HaloTag ligand. Protein concentration was determined by absorbance at 532 nm, the empirically determined absorption maximum based on NanoDrop measurements, and using an extinction coefficient of 94,000 M^−1^cm^−1^, based on prior characterizations of JF_525_ in hydrophilic environments [33]. From each conjugation batch, 4–5 fluorophore concentrations were prepared via direct dilution and used for subsequent measurements.

#### 4.1.4. Imaging Chamber Preparation

All samples were measured in individual wells of an 8-well chambered coverglass with a #1.5 borosilicate glass bottom (Nunc^TM^ Lab-Tek^TM^ II; Thermo Fisher Scientific, Waltham, MA, USA; cat. no. 155409). To minimize nonspecific surface adsorption, each well was incubated with 500 µL of 1% (*w*/*v*) bovine serum albumin (BSA) solution for at least 24 h at 4 °C. Immediately prior to sample addition, the BSA solution was aspirated, and each well was gently rinsed with deionized water to remove residual BSA and salts. Samples, including mCitrine protein solutions, JF_525_–HaloTag conjugates, and nanobead–Cygel mixtures, were then added directly to the wells for imaging.

### 4.2. Instrumentation and Data Acquisition

#### 4.2.1. Description of Microscope Setup

All fluorescence measurements were performed using a custom-built two-photon micro-spectroscope system (UW-Milwaukee Biophysical Microspectroscopy Facility, Milwaukee, WI, 53211), previously described in detail [38,51]. The instrument integrates a Zeiss Axio Observer inverted microscope stand with a C-Apochromat 63× water immersion objective with a numerical aperture (NA) of 1.2 (Carl Zeiss Microscopy, Oberkochen, Germany) and a highly sensitive electron-multiplying CCD (EMCCD) camera (iXon Ultra 897, Andor Technologies, Belfast, UK). This platform forms the core of the OptiMiS system, a configuration that has been employed across multiple previous fluorescence fluctuation spectroscopy and micro-spectroscopic studies [37,38,52].

Excitation was achieved using a single femtosecond-pulsed mode-locked Ti:Sapphire laser (MaiTai^TM^, Spectra-Physics, Milpitas, CA, USA), operated at a fixed wavelength of 960 nm. The excitation beam was expanded using a Galilean beam expander and then directed onto a phase-only spatial light modulator (SLM; Meadowlark Optics, Frederick, CO, USA; cat. no. P1920-1152-HDMI Nematic), which imposed spatially varying phase masks to shape the beam into a structured excitation pattern. For the protein solution measurements reported in this study, the beam was shaped into a 6 × 1 linear array of beamlets. Phase-mask modeling and beamlet formation procedures were consistent with previously established methods [38]. Galvanometric mirrors were positioned downstream of the SLM to enable precise control over beam positioning in the sample plane. For fluorescence solution measurements, these mirrors were held stationary to maintain a fixed excitation pattern during acquisition; for nanobead measurements, they were dynamically driven to raster the excitation array across the sample. All micro-spectroscope components, including the microscope stand, SLM, galvanometric mirrors, and EMCCD, were operated through custom control software written in C++ using Microsoft Visual Studio Community 2017 (Microsoft Corporation, Redmond, WA, USA).

#### 4.2.2. Data Acquisition: Fluorescent Nanobeads for Beam Waist Estimation

To characterize the spatial profile of the excitation beam and determine the virtual observation volume, 170 nm fluorescent nanobeads (PS-Speck, Invitrogen, Carlsbad, CA, USA; cat. no. P7220) were imaged on the same custom-built two-photon micro-spectroscope described in Section 4.2.1 using a two-dimensional (2D) x-y scanning configuration. Imaging was performed under excitation conditions that closely matched those used for protein solution measurements. Specifically, a 6 × 4 array of beamlets was generated using a spatial light modulator (SLM; Meadowlark Optics, Frederick, CO, USA; cat. no. P1920-1152-HDMI-Nematic; see Section 4.2.1), maintaining the same beamlet spacing as the 6 × 1 array used during protein measurements. This expanded array was raster-scanned across the sample using galvanometric mirrors, enabling parallel excitation over a wider field of view to capture a larger number of beads for improved statistical precision.

The lateral beam waist, $w_{0}$, was estimated at the focal plane (z=0 ) by analyzing the fluorescence intensity profiles of individual beads. For each selected bead, a line profile was drawn through the lateral center of the bead, and the fluorescence intensity was extracted as a function of pixel position. Because two-photon excitation produces a signal proportional to the square of the beam intensity, the square root of the fluorescence was analyzed to better reflect the beam profile. Pixel positions were converted to physical distances using a previously determined lateral calibration of 272 nm per pixel, determined from independent measurements. The resulting lateral intensity distributions were fit with one-dimensional Gaussian functions, and the 1/e^2^ width of each fit, $w_{fit}$, was extracted as a measure of the beam’s apparent width. These widths were then averaged across 15 beads to produce a mean lateral width representative of the excitation beam profile.

To account for the finite size of the 170 nm beads, which slightly broadens the measured width, a single deconvolution was applied to the average of the $w_{fit}$ values using the following formula [53,54]:

(4) $$w_{o}=\sqrt{{\bar{w}}_{fit}^{2}-w_{bead}^{2}}$$

Here, ${\bar{w}}_{fit}$ is the average 1/e^2^ width across all beads, and $w_{bead}$ is the effective waist of the fluorescent bead’s emission. The 170 nm nominal bead size was converted to a 1/e^2^ Gaussian width, $w_{bead}$, by dividing by the factor 1.177. This factor arises from the mathematical relationship between the full width at half maximum (FWHM) of a Gaussian distribution and its 1/e^2^ beam waist. This yielded a final corrected beam waist of $w_{o}=424\;\mathrm{nm}$, which was used in all downstream calculations of the excitation volume.

To determine the axial extent of the excitation beam, the same nanobead samples used for lateral profiling were imaged in a z-stack configuration. The microscope objective was incrementally stepped in 150 nm axial intervals through the bead, and a 2D fluorescence image was captured at each position. For each bead, the total fluorescence intensity within a 5 × 5 pixels region centered on the excitation spot was summed at every z-step. As with the lateral measurements, the square root of the integrated fluorescence was taken in order to account for the quadratic dependence of two-photon excitation on excitation intensity. The resulting axial intensity profiles were then fit with 1D Gaussian functions. To estimate the Rayleigh range ($z_{R}$) of the beam, we identified the axial position at which the square root of the signal dropped to half of its maximum value. This point corresponds to the axial distance at which the beam’s cross-sectional area has doubled, i.e., the physical point at which beam expansion dilutes the excitation flux by 50%. To ensure robustness, 21 beads were analyzed, and the resulting 1/e^2^ widths from individual beads were averaged and then converted to a Rayleigh length, yielding $z_{R}=703\;\mathrm{nm}$. While this experimentally determined value of 703 nm modestly exceeds the theoretical prediction (~600 nm) for a pure Gaussian beam with $w_{o}=424\;\mathrm{nm}$, we opted to use the measured value in downstream volume calculations.

#### 4.2.3. Data Acquisition: Fluorescent Solutions

All protein solution measurements were acquired using the fast kinetic series mode of the EMCCD camera. The excitation beamlets, formed by projecting a phase pattern onto the SLM (see Section 4.2.1), remained fixed during each acquisition as the galvanometric mirrors were held stationary, resulting in a stable 6 × 1 array of illumination volumes. For these measurements, the transmission grating in the OptiMiS detection head was removed, and the emission signal from each excitation voxel was collected without spectral separation.

Data were recorded using 2 × 2 binning applied along both rows and columns. The active imaging area was set to 10 binned rows. Each fast kinetic series acquisition consisted of 10,000 frames, with an exposure time of 100 µs per frame. After every 49 frames, the EMCCD array and the adjacent frame-transfer buffer were read out; this acquisition/readout process was repeated 205 times for a given time series The final dataset was saved as a 256 × 490 × 205 TIFF stack, where each 10-row block corresponded to a single time point in the 10,000-frame trace. The resulting dataset produced six independent intensity traces, one per beamlet, for subsequent brightness analysis. Twenty to twenty-five acquisitions were performed at different positions within the sample chamber to improve statistical sampling, resulting in 120–148 total intensity traces obtained from a given sample chamber.

### 4.3. Brightness Calibration via Virtual Volume Modeling

To relate measured fluorescence intensity to absolute molecular brightness under two-photon excitation, we modeled the spatial dependence of the excitation profile and defined a corresponding virtual observation volume. This approach accounts for the nonlinear excitation behavior and enables a direct, diffusion-independent estimation of brightness from intensity. In the context of two-photon excitation, where fluorescence scales quadratically with excitation intensity, the total signal from a uniformly distributed solution of fluorophores at concentration *C* can be expressed as [11,55]:

(5) $$I=\varepsilon ∭{PSF}^{2}\left(\vec{r}\right)C\left(\vec{r}\right)dV$$

Here, ε is the molecular brightness of a single fluorophore at the very center of the real beam. $PSF\left(\vec{r}\right)$ represents the spatial intensity variation in the actual laser beam. Notably, the quadratic dependence of signal on intensity in two-photon excitation leads to squaring this PSF when calculating fluorescence contributions, hence the ${PSF}^{2}\left(\vec{r}\right)$ in the volume integral. The integral over ${PSF}^{2}\left(\vec{r}\right)$ defines an effective or virtual observation volume, $V_{PSF}$, which effectively replaces the complex spatial weighting of the actual beam with an equivalent uniform volume, where all molecules are excited with maximal efficiency. The virtual observation volume is given by

(6) $$V_{PSF}=∭{PSF}^{2}\left(\vec{r}\right)dV$$

To model the spatial intensity distribution we use a Gaussian-Lorentzian hybrid PSF of the form [56]:

(7) $$PSF\left(\vec{r}\right)=\frac{w_{0}^{2}}{w^{2}\left(z\right)}e^{-2\left(\frac{x^{2}+y^{2}}{w^{2}\left(z\right)}\right)},$$

where $w_{0}$ represents the beam waist of the excitation beam (i.e., the 1/e^2^ radius of the beam at the focus), and $w^{2}\left(z\right)$ describes axial expansion of the beam along the optical axis, which can be modeled as

(8) $$w^{2}\left(z\right)=w_{0}^{2}\left[1+{\left(\frac{z}{z_{R}}\right)}^{2}\right]$$

where $z_{R}$ is defined as the Rayleigh length (or Rayleigh range) of the beam, in other words, i.e., the distance along the optical axis at which the cross-sectional area of the beam has doubled compared to the area at the center of the beam [57]. Equation (6) was numerically integrated in MATLAB R2024a (The MathWorks, Inc., Natick, MA, USA) using the measured values of $w_{0}=424\;nm$ and $z_{R}=703\;nm$, yielding $V_{\mathrm{PSF}}=0.28\;\mathrm{fL}$.

### 4.4. Data Reduction

Each single raw dataset was stored as a 256 × 490 × 205 TIFF stack, acquired under fast kinetic series mode (see Section 4.2.3). Each 10-row block represented a single time point in a 10,000-frame fluorescence time series. Within each block, six distinct intensity spots, corresponding to the six excitation beamlets, were present along the imaging array. To correct for baseline camera signal and stray light, background measurements were acquired daily with no sample on the stage. On average, five background time series were acquired per day, resulting in 1025 background images (256 × 490 pixels each). These were averaged into a single 256 × 490 pixels background frame and subtracted from each readout image in every single raw dataset prior to further processing.

For each time point in a single raw dataset, the pixel of maximum intensity was identified for each of the six excitation spots. A 5 × 5 square region was centered on this pixel, and the summed intensity within the square was taken as the fluorescence signal for that time point. Repeating this over all 10,000 time points produced six complete intensity traces, one per beamlet, for each single raw dataset. Multiple datasets were acquired per concentration condition to improve sampling statistics. For each intensity trace, the mean and variance of the signal were computed, and used to compute the effective molecular brightness for the given trace according to Equation (1). All effective brightness values corresponding to a given fluorophore concentration were compiled into a histogram and fit with a Gaussian function. The mean of the fitted Gaussian was taken as the effective molecular brightness for that sample.

## 5. Conclusions

This study presents a dual-method strategy for calibrating monomeric molecular brightness in fluorescence fluctuation spectroscopy (FFS), comparing traditional fluctuation-based estimates with an alternative approach based on average fluorescence intensity, known concentration, and a calibrated observation volume. We find that the intensity-based method yields more stable and consistent brightness estimates, particularly in aggregation-prone systems such as mCitrine, and is less sensitive to sample handling artifacts. While promising, this method depends on accurate concentration measurement and reliable volume calibration, and its applicability may be limited in heterogeneous or intracellular environments. Nonetheless, it offers a valuable cross-check on fluctuation-derived estimates and helps identify cases where aggregation compromises calibration accuracy. This method provides a practical route for determining monomeric brightness as a calibration step in FFS workflows. Future work will build on this framework by applying it to systems with known oligomeric states and exploring its use in benchmarking reversible self-association in solution.

## Figures and Tables

**Table 1:** Summary of mCitrine sample preparation conditions for indexed datasets. Sample groups varied in DTT content during FPLC elution, post-elution freezing conditions, PEG inclusion, and peak fractionation strategy. Index 2 represents a mixed group that includes overlapping fresh samples from Index 1 and flash-frozen samples with PEG. Indexes 5 and 6 highlight the impact of FPLC peak width and selection on apparent brightness measurements. The number of replicate measurements (N) used to calculate error bars in Figure 3 is listed in the second column.

Index	N	FPLC Elution Buffer	DTT (1 mM)	Storage Condition	PEG (1% *w*/*v*)	Notes
1	3	HEPES + 1 mM DTT	Yes	Fresh only	N/A	Reference condition
2	7	HEPES + 1 mM DTT	Yes	Mixture: fresh and flash-frozen	Yes (for frozen)	Subset overlaps with Index 1
3	3	HEPES + 1 mM DTT	Yes	Flash-frozen only	No	All post-thaw measurements
4	2	HEPES only (no DTT)	No	Flash-frozen only	No	Non-reducing buffer
5	5	HEPES only (no DTT)	No	Flash-frozen only	No	Subset rerun on FPLC; overlaps with Index 6
6	2	HEPES only (no DTT)	No	Flash-frozen only	No	Extremely broad (bimodal peak) on FPLC

## Data Availability

Dataset available on request from the authors.

## Supplementary Materials

The following supporting information can be downloaded at: https://www.mdpi.com/article/10.3390/ijms262311678/s1.
