# Supplementary material for: Intensity-Based Estimation of Monomeric Brightness for Fluorescent Proteins
Source: Int J Mol Sci. 2025 Dec 2;26(23):11678. doi: 10.3390/ijms262311678 (PMC12691995; doi:10.3390/ijms262311678)
Supplement: Supplementary file 1 [file ijms-26-11678-s001.zip › ijms-3931452-supplementary.pdf]

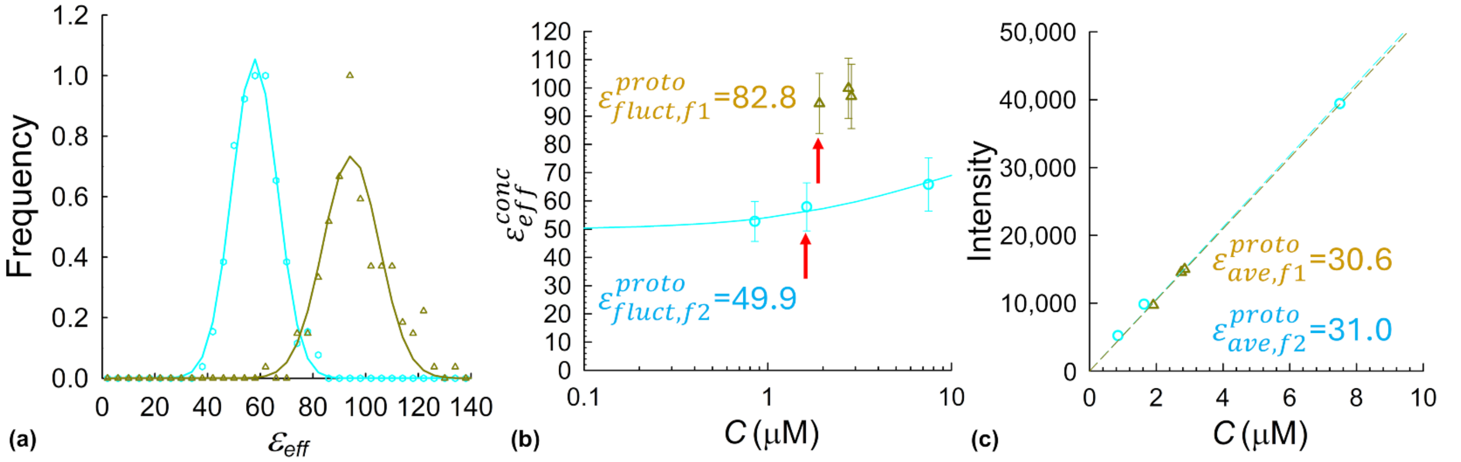

Supplementary Figure S1. Comparison of fluctuation-derived and average-intensity-derived molecular brightness for mCitrine from two FPLC-separated size fractions. Two preparations collected from the same purification run but separated by size-exclusion chromatography were analyzed: fraction 1 (f1), corresponding to a larger size fraction eluting earlier in the chromatogram (dark yellow, triangles), and fraction 2 (f2), corresponding to a smaller size fraction eluting later (cyan, circles). (a) Brightness histograms from representative measurements at similar concentrations (f1: 1.90 μM; f2: 1.63 μM), each computed from a collection of 10,000-frame fluorescence traces. Gaussian fits were used to extract the mean effective brightness for that concentration (ε_{eff}^{conc}). (b) Fluctuation-derived brightness values plotted as a function of fluorophore concentration for each fraction. The f2 data were fit using the monomer–dimer equilibrium model (Equation 2) to extrapolate the monomeric brightness, while the f1 data are shown without fitting for comparison. The red arrows point to the particular sample concentrations whose brightness histograms are shown in panel a. (c) Average fluorescence intensity plotted versus concentration for both fractions. The nearly overlapping linear fits yield slopes corresponding to intensity-based monomeric brightness estimates of 30.6 (f1) and 31.0 (f2) via Equation 8. Despite similar intensity-based estimates, f1 consistently exhibited higher fluctuation-derived brightness, indicating a greater proportion of higher-order species in the larger size fraction.

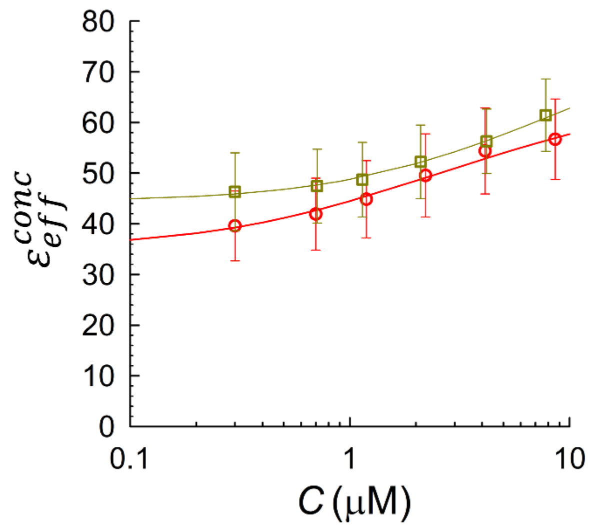

Supplementary Figure S2. Effect of sample handling on fluctuation-derived molecular brightness for mCitrine. Measurements were made from the same FPLC-purified preparation under two handling conditions: (1) immediately after purification (red circles) and (2) after aliquoting from the same fresh preparation, flash freezing without cryoprotectant, and thawing prior to measurement (dark yellow squares). Fluctuation-derived brightness values, ε_{eff}^{conc}., are plotted as a function of fluorophore concentration for each condition. Data for both handling conditions were fit with the monomer–dimer equilibrium model (Equation 2), with solid lines in the corresponding colors showing the fits. The fitted monomeric brightness values were $\varepsilon_{fluct}^{proto}$=34.3 for the fresh sample and $\varepsilon_{fluct}^{proto}$=44.4 for the freeze-thaw sample. The fresh sample shows lower brightness values across the measured concentration range, whereas the freeze–thaw sample exhibits systematically higher values, consistent with an increased proportion of aggregated species introduced by freeze–thaw without cryoprotectant.
